# Experimental Treatment of Hazardous Ash Waste by Microbial Consortium *Aspergillus niger* and *Chlorella* sp.: Decrease of the Ni Content and Identification of Adsorption Sites by Fourier-Transform Infrared Spectroscopy

**DOI:** 10.3389/fmicb.2021.792987

**Published:** 2021-12-07

**Authors:** Alexandra Šimonovičová, Alžbeta Takáčová, Ivan Šimkovic, Sanja Nosalj

**Affiliations:** ^1^Department of Soil Science, Faculty of Natural Sciences, Comenius University, Bratislava, Slovakia; ^2^Department of Environmental Ecology and Landscape Management, Comenius University, Bratislava, Slovakia

**Keywords:** microbial consortium, *Chlorella* sp., *Aspergillus niger*, nickel, FTIR spectroscopy, ash waste material

## Abstract

Despite the negative impact on the environment, incineration is one of the most commonly used methods for dealing with waste. Besides emissions, the production of ash, which usually shows several negative properties, such as a higher content of hazardous elements or strongly alkaline pH, is problematic from an environmental viewpoint as well. The subject of our paper was the assessment of biosorption of Ni from ash material by a microbial consortium of *Chlorella* sp. and *Aspergillus niger*. The solid substrate represented a fraction of particles of size <0.63 mm with a Ni content of 417 mg kg^–1^. We used a biomass consisting of two different organisms as the sorbent: a non-living algae culture of *Chlorella* sp. (an autotrophic organism) and the microscopic filamentous fungus *A. niger* (a heterotrophic organism) in the form of pellets. The experiments were conducted under static conditions as well as with the use of shaker (170 rpm) with different modifications: solid substrate, *Chlorella* sp. and pellets of *A. niger*; solid substrate and pellets of *A. niger*. The humidity-temperature conditions were also changed. Sorption took place under dry and also wet conditions (with distilled water in a volume of 30–50 ml), partially under laboratory conditions at a temperature of 25°C as well as in the exterior. The determination of the Ni content was done using inductively coupled plasma optical emission spectrometry (ICP-OES). The removal of Ni ranged from 13.61% efficiency (*Chlorella* sp., *A. niger* with the addition of 30 ml of distilled water, outdoors under static conditions after 48 h of the experiment) to 46.28% (*Chlorella* sp., *A. niger* with the addition of 30 ml of distilled water, on a shaker under laboratory conditions after 48 h of the experiment). For the purpose of analyzing the representation of functional groups in the microbial biomass and studying their interaction with the ash material, we used Fourier-transform infrared (FTIR) spectroscopy. We observed that the amount of Ni adsorbed positively correlates with absorbance in the spectral bands where we detect the vibrations of several organic functional groups. These groups include hydroxyl, aliphatic, carbonyl, carboxyl and amide structural units. The observed correlations indicate that, aside from polar and negatively charged groups, aliphatic or aromatic structures may also be involved in sorption processes due to electrostatic attraction. The correlation between absorbance and the Ni content reached a maximum in amide II band (*r* = 0.9; *P* < 0.001), where vibrations of the C=O, C–N, and N–H groups are detected. The presented results suggest that the simultaneous use of both microorganisms in biosorption represents an effective method for reducing Ni content in a solid substrate, which may be useful as a partial process for waste disposal.

## Introduction

In view of the relatively high consumption of natural resources and energy, finding alternative methods of obtaining renewable sources of raw materials or waste recycling has become a priority for every developed country (Markoš et al., 2010). Waste, along with the prevention of waste generation and recycling, is coming to the attention of the environmental policy of European Union Member States. The current policy of EU Member States stakes out three basic goals: (1) preventing waste generation, (2) supporting waste reuse by recycling, (3) recovering waste to reduce its impact on the environment (Directive 86/278/EEC, 1986; Directive 91/271/EEC, 1991; Directive 2000/76/EEC, 2000; Geffert and Geffertová, 2004; Geffert and Mojžišková, 2009).

With the rapid growth of industrialization worldwide, risk elements (cadmium, lead, nickel, cobalt, copper, zinc, and chromium) are being generated into wastewater. These are toxic and non-biodegradable pollutants with high toxicity even at very low concentrations. They accumulate in the food chain and are adsorbed in organisms, a result of which is serious health problems (Nebeská et al., 2018; Sathe et al., 2018; Kushwaha et al., 2019).

Removing heavy metals from wastewater through the individual steps of a wastewater treatment plant is insufficient, and these metals are subsequently accumulated in the treatment plant sludge. Wastewater treatment plants produce three types of sludge: primary sludge, excess activated sludge, and digested sludge. Primary sludge is generated by precipitation of insoluble substances and organic compounds, while excess activated sludge is a result of the subsequent biological treatment system (aerobic or anoxic-oxic) (Pinto et al., 2016). The primary sludge is taken from the settling tanks and mixed with the concentrated excess biological sludge. This mixed sludge is anaerobically treated in digesters. After anaerobic stabilization of the raw sludge, digested sludge is formed, which is less hazardous considering hygienic standards.

Sludge contains >95% water, so it must be drained before disposal. Although sludge is rich in nutrients, its application in agriculture is strictly regulated due to the presence of pollutants and pathogens. In this regard, its incineration is often a preferred method of recovery. The advantage of incinerating sludge is the processing of a large volume of waste, while the negative side of this process is the production of gas emissions and also solid waste–ash.

Sludge ash, as a waste material, often contains several risk elements, including mercury, arsenic, chromium, selenium, nickel, barium, and manganese. Besides alkaline pH, a characteristic attribute of ash is that the elements in it are present mainly in the form of oxides. Some of these oxides are characterized by significant solubility in water, which is associated with a higher risk of mobilization of selected elements. On the other hand, the properties of the ash can be exploited in uses with various added value, from low to technologically advanced applications, thus bringing substantial economic benefits. Ash is used, for example, for soil reclamation, in construction, in the ceramics industry, in catalysis processes, in the synthesis of zeolite and for precious metals regeneration (Dai et al., 2012; Li et al., 2018; Munir et al., 2018). However, as reported by Ukwattage et al. (2013), the direct application of ash for increasing the yield of crops due to the high content of micro- and macronutrients is limited due to its categorization as hazardous waste from a legislative point of view. Disposal of ash by solidification, which at present is used currently, is only a partial solution, because by changing ash into a stabilized form, a possible secondary raw material containing useful components is irreversibly lost.

Ash generated from a sludge incinerator offers high potential for heavy metal recycling (Reijnders, 2005). Several techniques have been tested to reduce contamination. The most common are: decomposition treatment with the addition of inorganic acids; phosphoric acid stabilization (Vavva et al., 2017), elution with nitric acid (HNO_3_) (Wang Y. et al., 2015), application with diluted sulfuric acid (H_2_SO_4_) (Kashiwakura et al., 2010), washing with hydrochloric acid (HCl) (Weibel et al., 2018); using citric acid for stabilization (Wang H. et al., 2018), using chelating agents for electroplating processes (Chen et al., 2015), and using hydrothermal processes (Ullah et al., 2018).

The use of a biosorbents based on different types of biomass, including bacteria, fungi, algae, yeast, and aquatic plants, has been studied in the process of nickel biosorption. According to Sari and Tuzen (2008) and Wang and Chen (2009), biosorption onto living or non-living biomass, can be a feasible method for metal removal, because it is efficient, minimizes secondary wastes and utilizes low-cost materials (Montazer-Rahmati et al., 2011). Algae are among the excellent sorbents due to their high relative binding capacity. Sorbents such as activated carbon and natural zeolite are more effective, and the results of sorption are comparable to the ion exchange processes that take place using resins. In case of algal biomass various structural units are involved in the adsorption including carboxyl, amino and hydroxyl groups in algal cell-wall polysaccharides, which can act as binding sites for metals (Guarín-Romero et al., 2019). There is evidence that phenomena of ion exchange, chelation, inorganic precipitation, or a combination thereof occur at the cell membrane of microorganisms (de Rome and Gadd, 1991; Tiemann et al., 1999; Deng et al., 2007).

In recent studies biosorption processes were often assessed with simultaneous use of various organisms in form of microbial consortia. The microscopic green algae *Chlorella* sp. and the microscopic filamentous fungus *Aspergillus niger* were applied in wastewater treatment (Nasir et al., 2019). Removal of cadmium by consortium of *A. niger* and *Chlorella vulgaris* (Bodin et al., 2017) and removal of arsenic by *Aspergillus oryzae* and *C. vulgaris* (Li et al., 2019) in wastewater treatment were confirmed. Raval et al. (2016) reviewed a variety of adsorbents for the removal of nickel(II) ions from wastewater using activated charcoal but also a biomass of the green algae *C. vulgaris*, brown algae, such as *Nizmuddinia zanardini, Sargassum glaucescens, Cystoseria indica*, and *Padina australis* and filamentous fungi, such as *A. niger* and *Rhizopus nigricans*. The species *Rhizopus stolonifer* proved to be effective in the removal of lead, cadmium, copper, and zinc from contaminated soil or a solid substrate (Fawzy et al., 2017), and *Mucor* sp. together with the microalgae *Chlorella* sp. have also been widely used in water treatment. The mycoalgae biofilm based processes, propounds the scope for exploring new avenues in the bio-production industry and bioremediation (Rajendran and Hu, 2016). The coagulation of microscopic algae and filamentous fungi in the form of pellets has many advantages (such as harvesting by simple filtration) and is also very advantageous economically because of the low costs (Alrubaie and Al-Shammari, 2018).

The aim of our work was to evaluate the use of a consortium of two different microorganisms–*Chlorella* sp. (autotrophic) and *A. niger* (heterotrophic)–as a tool for decreasing the nickel content in hazardous ash waste from an incinerator. Most of the work that has focused on the assessment of the biosorption of metals by a microbial biomass used only a single organism in the experiments conducted. In this work, we decided to use *A. niger* and *Chlorella* sp. at the same time for Ni(II) ions sorption by consortium biomass. We started with the assumption that if the adsorbent is formed by a mixture of biomass of both organisms, it will result in a greater diversity of its chemical composition, which may in the end lead to a larger amount of adsorbed Ni. In the framework of studying decrease of the nickel content, we focused on identifying the structural units of the biomass of the given microorganisms that Ni binds to during sorption. Similar to other studies, we also used FTIR spectroscopy for this purpose. In works with a similar focus, the authors typically interpreted changes in the spectrum of the biomass sample before and after its interaction with the hazardous element (Pradhan et al., 2007; D’Souza et al., 2008; Kang et al., 2011; Guarín-Romero et al., 2019). A change in the peak parameters usually helped to identify the functional groups bound in the biomass that are involved in the retention of the element. In this study we used slightly different approach. We identified the given functional groups on the basis of a correlation spectrum, where each value represents the Pearson correlation coefficient expressing the collinearity between the absorbance at a given wavelength and the adsorbed Ni content for a selected number of samples. We assume that in this way the main sorption sites of microbial biomass can be accurately identified.

## Materials and Methods

### Ash Substrate for the Disposal of Waste (Nickel)

**FIGURE 1:**
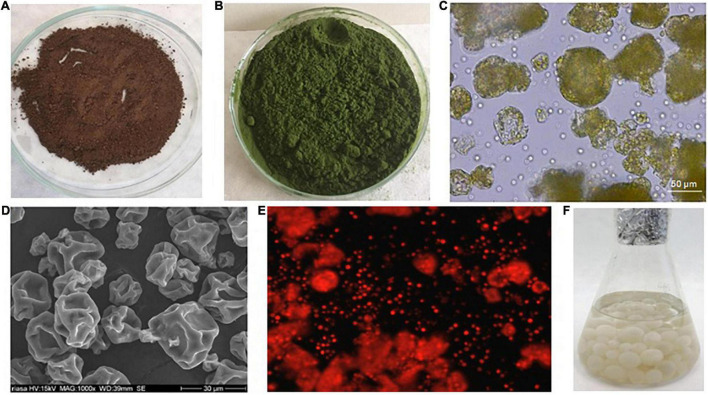
**(A)** Treated ash substrate from the sludge incinerator. **(B)** Dry biomass of the green alga *Chlorella* sp. after pre-treatment. **(C)** Dry biomass of the green alga *Chlorella* sp. in a light microscope. **(D)** Dry biomass of the green alga *Chlorella* sp. in SEM. **(E)** Dry biomass of the green alga *Chlorella* sp. in a fluorescence microscope. **(F)** Pellets of *Aspergillus niger* after washing in distilled water.

The ash intended for nickel decontamination comes from an industrial sludge incinerator (Figure 1A). The ash was analyzed on the basis of a requirement for landfill waste recovery (Table 1). The sample was taken as a so-called mixed sample, i.e. from ten partial point samples from a temporary storage site (a landfill), namely so that all properties of the waste are representative. The pre-treatment consisted of quartation, homogenization and subsequent fractionation through a fractional sieve (mesh size 0.63 mm).

**TABLE 1:** Input values of ash from the sludge incinerator.

Parameter	Determined value	Unit of dry weight
Hg	0.02	mg kg^–1^
As	41.0	mg kg^–1^
Cd	7.64	mg kg^–1^
Ni	417	mg kg^–1^
Pb	146	mg kg^–1^
PAU	<0.1	mg kg^–1^
P	5.88	% (w/w)
S	1.22	% (w/w)
C	3.43	% (w/w)
H	0.36	% (w/w)
N	0.32	% (w/w)
NEL	<10.0	mg kg^–1^
TOC	0.86	% (w/w)
dry mass	98.0	% (w/w)

*PAU, polyaromatic hydrocarbons; NEL, non-polar extractables; TOC, total organic carbon.*

### Analysis of Metals in the Ash Substrate

The basis of the method is the measuring of the atomic emission by optical spectroscopy. After mineralization (decomposition of solid samples into an aqueous matrix), the samples are sucked using a peristaltic pump through a nebulizer into a misting chamber, where they are nebulized. The aerosol created is then carried into a plasma, where excitation of the atoms occurs. Characteristic atomic line spectra are then generated by high frequency inductively coupled plasma (ICP). The radiation emitted is decomposed using a spectrometer grid and the intensities of the lines are monitored by detectors.

The determination of metals for pre-treatment of the ash took place by extraction under a reflux condenser. The original homogenized sample was weighed in the amount of 0.3 g into a reaction vessel and 21 ml of HCl and 7 ml of HNO_3_ were added.

The reaction vessels were left open until the initial reaction (foaming) took place. The mixture was covered with a watch glass and left to stand at room temperature for 16 h. The vessels were subsequently placed on a heater and the reflux condenser was installed. The temperature of the reaction mixture was slowly raised until reflux conditions were reached. This state was maintained for 2 h to ensure that the cooling zone is lower than one-third the height of the reflux condenser. The reaction mixture was then allowed to cool. The condenser was rinsed with 10 ml of 1 mol L^–1^ nitric acid in the reaction vessel. The mineralizate was then poured quantitatively through a filter paper into a 100 ml measuring cup, filled up to the mark and then poured into a 100 ml plastic storage container.

### Green Algae *Chlorella* sp. as the Autotrophic Organism

The genus *Chlorella* sp. as a representative of algae ranks among the most numerous groups of green algae. In all the experiments an algal biomass of *Chlorella* sp. obtained from Institute of Microbiology of the Czech Academy of Sciences (Opatovický mlýn, Tr̆ebon̆, Czechia), was used (Figures 1B–D). The dry biomass of algae was prepared by washing the biomass in deionized water and drying in an oven at 70°C for 24 h.

### Microscopic Filamentous Fungus *Aspergillus niger* as the Heterotrophic Organism

**FIGURE 2:**
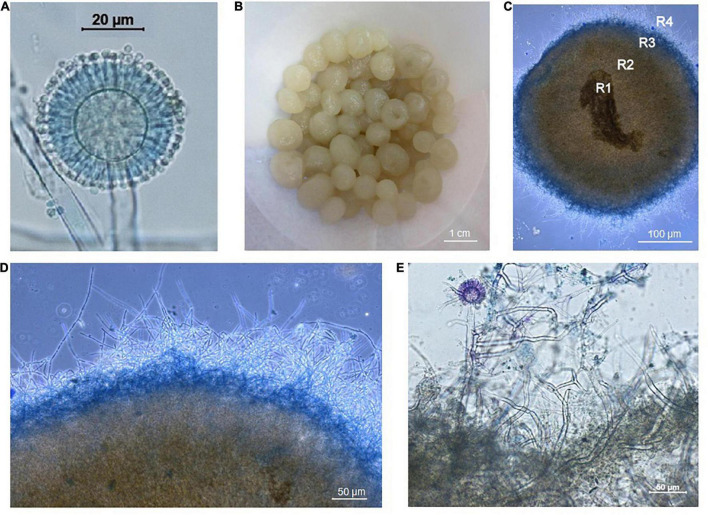
**(A)** Conidial head of *Aspergillus niger* (conidiophore terminated by a vesicle with phialides and conidia). **(B)** *Aspergillus niger* pellets after washing and filtration. **(C)** *Aspergillus niger* pellets, which have four visible parts according to García-Reyes et al. (2017). R1 is the core of the pellet, R2 the hollow part, which may be around the core, R3 is the layer of fibers which may show signs of autolysis, R4 is the outer part of the pellet which appears to be fuzzy or hairy, the so-called “hairy region” and consists of viable fibers. **(D)** Detail of the “hairy region” of the pellet. **(E)** Detail of “hairy region” of the pellet with a conidiophore of *Aspergillus niger*.

A cosmopolitan strain of *A. niger* (Figure 2A) was isolated from Dystric Cambisol (contaminated and eroded) without vegetation. The chemical characteristics of the substrate showed an ultra-acidic soil reaction (pH 3.12), a very low amount of organic matter (%C_ox_ 0.49) and exceeded the value of aluminum (Al 727 mg kg^–1^) (Šimonovičová et al., 2021).

*Aspergillus niger* fungal pellets were prepared in a 45 ml of SDB (Sabouraud Dextrose Broth Liquid Medium, Himedia, Mumbai, India) enriched with a 5 ml suspension of conidia from a pure culture of *A. niger*. Cultivation took place over 5 days at 25°C in 250 mL Erlenmeyer flasks, with stirring at 170 rpm (Unimax 2010 shaker, Heidolph, Germany) under laboratory condition at 25°C (Figure 1F). The *A. niger* fungal pellets formed very quickly, and after 5 days they were removed by filtration, washed with a large amount of distilled water and used in the experiments (Figure 2B).

### Microscopy

**FIGURE 3:**
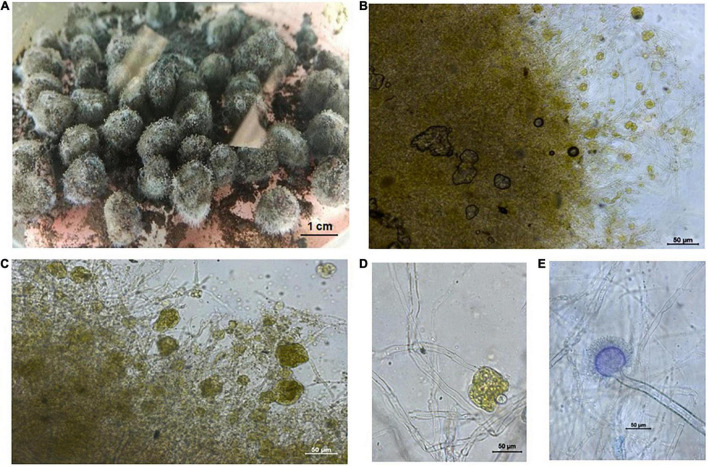
**(A)** *Aspergillus niger* pellets after application to the ash substrate under laboratory conditions on a shaker and without addition of water. **(B)** Detail of the *Aspergillus niger* pellet with *Chlorella* sp. and with nickel particles. **(C,D)** Detail of the “hairy region” of the *Aspergillus niger* pellet with *Chlorella* sp.. **(E)** Detail of “hairy region” with the conidial head of *Aspergillus niger*.

The figures of the dry green algae biomass (Figure 1C), the filtered *A. niger* pellets (Figures 2C–E, 3B–E), were made with a Canon IXUS 16.1 megapixel camera (Japan).

The figures of the *Chlorella* sp. surface structure (Figure 1D) were observed under a JEOL JXA 840A Scan Electron Microscopy (SEM) (Japan).

The figures of the *A. niger* strain (Figure 2A), the *A. niger* pellets (Figures 2C–E), and the *A. niger* with *Chlorella* sp. pellets (Figures 3B–E) were observed under an Axio Scope A 1 Carl Zeiss Jena light microscope in a drop of lactic acid enriched with a cotton blue stain (0.01%).

### Fluorescence Recording

Microscopic measurements [Microscope: AXIO–IMAGER A, CARL ZEISS (Germany), Filter set 02–excitation G 365, Beam splitter FT 396, Emission LP 420], chlorophyll in a metabolically inactive biomass (Figure 1E).

### Fourier-Transform Infrared Spectroscopy

In the case of each sample, approximately 1 g of bulk material was pulverized into a fine powder in an agate mortar. After homogenization, the samples were dried at 60°C for 24 h. Before each measurement 2 mg (± <0.05 mg) of homogenized sample was thoroughly mixed with 200 mg of KBr and pressed into a small pellet. FTIR analysis was performed in transmission mode and the spectral data were expressed as absorbance values. Each spectrum was recorded in the mid-infrared region (from 4000 to 400 cm^–1^) by averaging 128 scans, with a spectral resolution of 4 cm^–1^. The analysis was performed using a NICOLET 6700 FTIR spectrometer and the OMNIC 8 software (Thermo Scientific). The FTIR spectra obtained were not corrected by any procedures, filters, or algorithms, such as smoothing, absorbance normalization, baseline corrections, etc.

### Elemental Analysis (CHNSO)

Mass concentrations of C, H, N, and S were determined by combustion of the sample (app. 10 mg) in the atmosphere with elevated oxygen content and subsequent detection of liberated gases by the gas chromatography principle. The O content was measured similarly, but the sample was heated in an atmosphere of pure He, without the addition of a catalyst. The measurement was carried out by the elemental analyzer FLASH 2000 equipped with the Eager Xperience software (Thermo Scientific).

### Organization of Experiments

All the experiments were organized according to Table 2. In all samples 3 g of ash were used. Dry biomass of *Chlorella* sp. in an amount of 1 g was used only in samples 5–15. *A. niger* fungal pellets were also used in all samples. Distilled water in a volume of 30 ml was added to samples 1, 3, 5, 6, 9, 10, and a volume of 50 ml was added only to sample 15. Samples 1, 3, 5, 6, 13, and 15 were performed on a shaker, while all other samples were run under static conditions.

**TABLE 2:** Conditions of all experiments in laboratory or in the exterior.

Sample	Ash (3 g)	Pellets of *A. niger*	*Chlorella* sp. (1 g)	Distilled water	Shaker 170 rpm/min.	Laboratory exterior
1	+	+	−	30 ml	+	L
2	+	+	−	−	−	L
3	+	+	−	30 ml	+	L
4	+	+	−	−	−	L
5	+	+	+	30 ml	+	L
6	+	+	+	30 ml	+	L
7	+	+	+	−	−	L
8	+	+	+	−	−	L
9	+	+	+	30 ml	−	E
10	+	+	+	30 ml	−	E
11	+	+	+	−	−	E
12	+	+	+	−	−	E
13	+	+	+	−	+	L
14	+	+	+	−	−	E
15	+	+	+	50 ml	+	L

*L, laboratory; E, exterior.*

We chose the variability of individual samples within the experiments based on the different decontamination conditions both under natural conditions (exterior) and under laboratory conditions at a temperature of 25°C. The samples for analysis were taken at a defined time interval of 48 h. At the end of the experiments in the case with the addition of distilled water, the suspension was separated from the solution by membrane filtration (Millipore, pore size 0.45 μm). The metal analysis of all samples was carried out on an emission spectrometer with inductance coupled plasma (ICP Profile Plus, Teledyne Leeman Labs, United States). All experiments were performed in duplicate.

## Results and Discussion

### The Role of the Microbial Consortium in the Decrease of Ni Content

**FIGURE 4:**
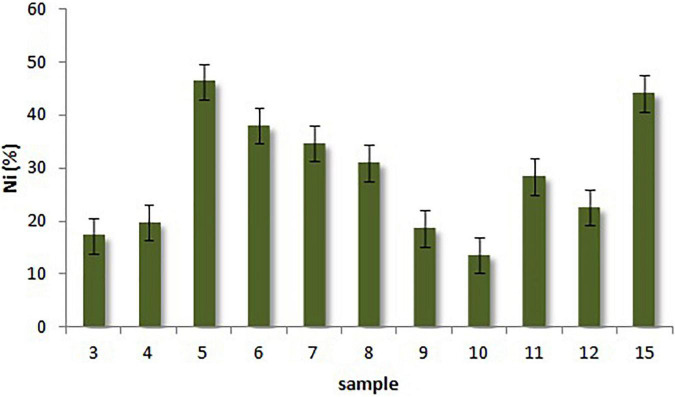
Decrease of the nickel content in the ash from the sludge incinerator by the microbial consortium.

The input values of the ash from the sludge incinerator are documented in Table 1. The removal of Ni from the material ranged as follows: 13.61% (sample 10) < 17.25% (sample 3) < 18.58% (sample 9) < 19.68% (sample 4) < 22.49% (sample 12) < 28.37% (sample 11) < 30.95% (sample 8) < 34.56% (sample 7) < 37.90% (sample 6) < 44.01% (sample 15) < 46.28% (sample 5), as shown in Figure 4.

From the various experimental conditions, the combination of *A. niger* pellets and dry algal *Chlorella* sp. biomass, with the ash substrate moistened with 30 ml to 50 ml of distilled water, proved to be the most effective. The experiment ran on a shaker under laboratory conditions. During shaking, in addition to the even distribution of pellets with the ash substrate, the coagulation of pellets with the algal biomass also occurred (Figures 3A–E). The coagulates formed not only absorbed Ni on the surface but also bioleached in the presence of *A. niger* (samples 5, 6, and 15).

Under laboratory conditions, when only *A. niger* pellets were applied to the ash, we recorded a slightly higher efficiency under static conditions and without the addition of distilled water (sample 4) compared with the addition of 30 ml of distilled water on a shaker (sample 3). In the exteriors, experiments were conducted under static conditions, and in this case, too, we recorded lower values of Ni content reduction in the aqueous medium (samples 9 and 10) compared with the anhydrous environment (samples 11 and 12). The species *A. niger* is a known producer of many organic acids and secondary metabolites (Šimonovičová et al., 2020, 2021) which are used in bioleaching processes. It can be assumed that in the case of adding distilled water, the organic acids produced were diluted, which caused a slowing down/reduction of the bioleaching process from the solid substrate.

The absorption of Ni from the ash substrate occurs by means of several processes. When *A. niger* pellets are used alone, the process of bioleaching takes place. When a consortium of the microorganisms *Chlorella* sp. and *A. niger* were used, aside from the bioleaching, the efficiency is increased by involving the cell wall of green algae biomass. The cell wall of algae is made up of cellulose microfibrils, pectins, hemicelluloses, and proteins. These polymers form a complex network of pores and channels that influence the movement of substances in this space. But the surface negative charge of polymers can limit the movement of minerals as well as heavy metals by binding them. However, in general, the cell wall is permeable. The cytoplasmic membrane, as well as the vacuole membrane, represent a highly selective semipermeable barrier between the internal and external cell environment, and its role is to control the transfer of substances into and out of the cell to the surrounding space. It is only permeable for certain substances, for example, the molecules of some gases (O_2_, CO_2_, N_2_) and for water, but less so for glycerol or ethanol. For substances that cannot pass freely into the cell through the cytoplasmic membrane, transmission is provided *via* pumps, transporters, and channels (which are membrane proteins), which are more or less specific for transport of the given substance.

For us, the so-called ABC carriers, which is a general term used for a large and diverse group of carriers, are of interest. ABC transporters on a tonoplast provide transport, for example, of heavy metals or herbicides into the vacuole as well as their sequestration. In an environment where different ions are present, competition often occurs between, for example, Ca^2+^ and other cations at the interaction site. Thus, instead of a “good cation,” an unwanted ion, e.g., Cd or ion of another heavy metal, enters the cell. However, with damaged cells and a disrupted cell membrane system a loss of membrane semipermeability and uncontrolled movement of substances occurs. Microorganisms have the ability to accumulate many heavy metals and toxic elements (Pb, Ag, Pt, Pd, Au, Hg, Ga, Cd, Cu, Ni) from the external environment (Bulgariu and Bulgariu, 2020). Living and dead cells, the products of cell metabolism, extracellular polysaccharides and cell wall components all have the ability to receive and accumulate metals (Timková et al., 2018). The primary carrier surface of biosorption, however, is the cell wall of the biosorbent. The composition of the cell wall consists particularly of polymeric substances that are rich sources of various functional groups, by which the contaminants bind. The following groups can be included here: carboxyl (–COOH), hydroxyl (–OH), sulfhydryl (–SH), phosphate (–PO_4_^3–^), amino (–NH_4_^+^), and others (Gupta and Diwan, 2017).

The metabolically inactive, i.e., the dead culture of an algal biomass, can isolate metal ions and metal complexes from solution due to its unique chemical composition. When selecting metal-sorbent biomaterials, it is necessary to start from the factor of origin and thus resolve the issues of availability, quantity and potential demand (de Rome and Gadd, 1991; Tiemann et al., 1999). The advantage of applying dead biomass compared to a living one, i.e., an active biomass, is that the living biomass cells require the addition of a fermentation medium (which are costly), and the simultaneous addition of medium increases biological oxygen demand (BOD) and chemical oxygen demand (COD). Furthermore, dead biomass is not affected by the toxicity of metal ions and can be subjected to various chemical and physical pre-treatments to increase sorption. As a result, adsorbed metals can be easily obtained from the biomass by chemical or physical methods, which leads to repeated use of the biomass, thus reducing the costs of the process. Ash classified as hazardous waste is disposed of by several methods, such as heat treatment (e.g., sintering, melting and vitrification) (Park et al., 2005), the setting of cement (Bie et al., 2016), chemical stabilization (Nzihou and Sharrock, 2002; Wang F. H. et al., 2015), or extraction (Tang and Steenari, 2015; Wang W. et al., 2018). Other alternative methods for reducing hazardous substances in ash, which run with a lower energy load or better performance, include hydrothermal treatment (Jin et al., 2013; Qiu et al., 2017, 2018) and mechanical-chemical treatment (Chen et al., 2019).

### Fourier-Transform Infrared Spectra of *Chlorella* sp. and *Aspergillus niger* Biomass

There were several differences between the spectra of *Chlorella* sp. and *A. niger*, respectively, which are related to the different chemical composition of the two microorganisms. According to Johnson (1965), the main components building the mycelium of *Aspergillus* are carbohydrates (73–83%), with smaller portions of lipids (2–7%) and proteins (0.5–2.5%), while in the case of *Chlorella* sp., approximately 50% of the biomass is made up of proteins (Wild et al., 2019). Apart from proteins, *Chlorella* sp. Also contains a higher amount of lipids (∼18%) and a significantly lower concentration of carbohydrates (∼23%) in comparison to *A. niger* (Prabakaran et al., 2018). The results of FTIR spectroscopy were in general accordance with these differences. IR absorption in the *Chlorella* sp. spectrum may be associated mainly with amides and aliphatic structures, whereas the spectrum of *Aspergillus* showed more peaks characteristic for carbohydrates (polysaccharides). It should be noted that amide groups are not only a part of proteins but are also present in certain polysaccharides, such as chitin. In some cases, the amides bound in different structural units can be distinguished, as the vibrations of the respective functional groups manifest at different frequencies. The nitrogen content detected in the biomass was lower in the case of *A. niger* in comparison to *Chlorella* sp., and the same accounts for the N/C ratio (Table 3). This is associated with total amount of proteins in the biomass, which was probably lower in the case of *Aspergillus*.

**TABLE 3:** Organic elemental composition (CHNSO) of biomass samples and solid substrate together with contents of adsorbed Ni.

Sample	C	H	N	S	O	SUM	C/N	Ni
	g kg^–1^							mg kg^–1^
1	402.80	62.71	56.62	2.76	398.23	923.11	7.11	–
2	390.76	60.95	57.34	1.44	400.88	911.37	6.82	–
3	57.73	6.43	6.10	1.55	136.56	208.36	9.47	55
4	109.10	13.46	13.28	5.39	165.31	306.53	8.22	62
5	175.74	24.72	27.50	5.72	207.08	440.75	6.39	147
6	135.43	15.88	22.05	2.08	151.86	327.30	6.14	120
7	118.31	13.80	18.83	1.22	146.53	298.68	6.28	110
8	149.87	18.44	23.94	5.71	174.13	372.08	6.26	98
9	160.58	19.61	24.29	–	166.40	370.88	6.61	59
10	167.06	20.80	27.09	2.15	168.08	385.18	6.17	43
11	165.54	22.98	26.74	–	192.37	407.63	6.19	24
12	168.97	23.33	27.29	5.08	190.74	415.40	6.19	71
13	452.89	66.08	75.23	3.78	340.55	938.53	6.02	–
14	506.22	64.74	62.63	–	320.15	953.75	8.08	–
15	231.55	30.56	38.80	2.72	217.88	521.52	5.97	140
*Chlorella* sp.	486.77	67.16	95.13	19.52	303.64	972.21	5.12	–

*Samples 1–15 are assigned according to Table 2.*

**FIGURE 5:**
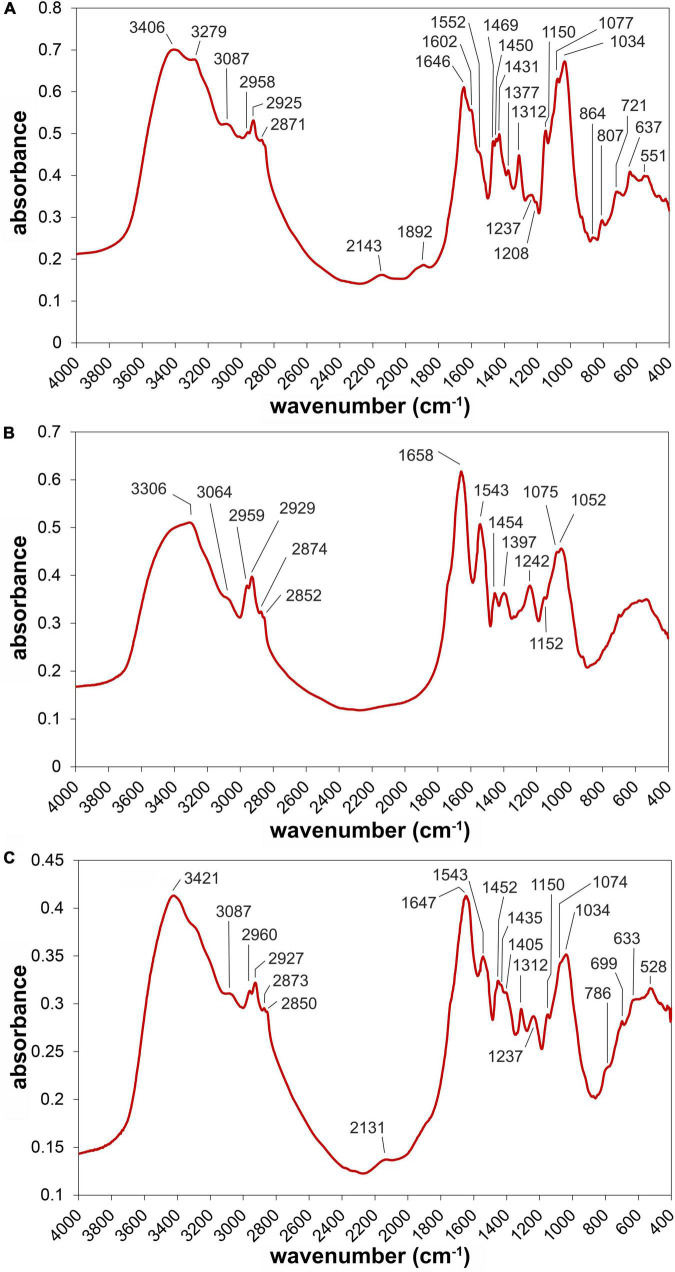
FTIR spectra of *Aspergillus niger* biomass **(A)**, *Chlorella* sp. **(B)**, and the consortium of both microorganisms **(C)**, respectively.

There was a peak around 3280 cm^–1^ in the *A. niger* spectrum (Figure 5A) which was much less visible in the case of *Chlorella* sp. (Figure 5B). This absorption may be assigned to amines, which were detected in various fungal species and strains (Stüttgen et al., 1978; Karimi et al., 2019). Johnson (1965) reported that the cell wall of *A. niger* contains approximately 9–13% hexosamines. Amine and carboxylate groups were identified as major functional groups for metal biosorption in the *A. niger* biomass (Cai et al., 2016). This is in accordance with results presented here, as one of the peaks of the correlation spectrum reached a maximum at 3284 cm^–1^, which lies within the amine frequency interval. Specifically, IR absorption around 3280 cm^–1^ is caused by the N–H stretching of secondary amines (Smith, 2019). However, this peak may also be associated with the amides bound in proteins (Forfang et al., 2017) or chitin (Rinaudo, 2006), as stretching vibrations of the N–H group and associated absorption around 3280 cm^–1^ are characteristic for both of these components. The peak with a maximum at 2143 cm^–1^ in the *A. niger* spectrum can be associated with isocyanides, which have been identified in various microorganisms, including *Aspergillus* species (Massarotti et al., 2021). According to Bittante and Cecchinato (2013), isocyanides show characteristic absorption between 2110 and 2165 cm^–1^.

Another difference observed in the two spectra is the peak at 1602 cm^–1^, which was absent in case of *Chlorella* sp. The absorption at frequencies around 1600 cm^–1^ is associated with vibrations of the aromatic ring and the presence of melanin pigments in the fungal biomass (Meenu and Xu, 2019). Information on the synthesis of various pigments by filamentous fungi, including *Aspergillus* species, were reviewed by Kalra et al. (2020). Significant differences between the spectra of *A. niger* and *Chlorella* sp. were detected in the regions of amide vibrations, particularly in the amide I and II spectral bands. This is related mainly to the differing overall protein content in the biomass of the two compared microorganisms, which was probably significantly higher in the case of *Chlorella* sp. Amide I and II bands are typically represented by two distinct peaks with maxima at around 1656 and 1547 cm^–1^, respectively (Lorenz-Fonfria, 2020). These two peaks are very apparent in the *Chlorella* sp. spectrum, whereas they are less prominent in the case of *A. niger*. The absorption in each of the two amide bands is associated with the combination of vibrations of several functional groups. The amide I peak results from the stretching of the C=O and C–N groups, respectively, as well as N–H bending. Amide I absorption is primarily caused by the C=O stretching vibration of the amide group, with weaker contributions from the amide C–N stretching and the N–H bending. In the case of the amide II band, the absorption is caused mainly by N–H bending and C–N stretching (van Hoogmoed et al., 2003; Lorenz-Fonfria, 2020). However, due to the complex and variable structure of proteins, slightly differing frequencies are reported for the amide I and amide II bands when data from various sources are compared.

The spectra of *A. niger* and *Chlorella* sp. also exhibited different absorption patterns between 1470 and 1430 cm^–1^. Whereas the spectrum of *Chlorella* sp. showed only one peak (1454 cm^–1^) in the considered interval, the spectrum of *A. niger* showed a triplet of peaks, with maxima at 1469, 1450, and 1431 cm^–1^, respectively. There are two adjacent bands in which the vibrations of aliphatic structures are detected: asymmetrical CH bending in the methyl group at 1470 cm^–1^ and methylene scissoring at 1465 cm^–1^ (Stuart, 2004). Other works report slightly different frequencies for these two bands. For example, Hollas (2004) associates the 1444 cm^–1^ frequency with asymmetric deformations of CH_3_, while Haneef et al. (2017), who used fungal mycelia as a source of various fibrous materials, linked symmetric bending of CH_2_ with the absorption around 1450 cm^–1^. Although various authors who analyzed a fungal biomass associated the peaks between 1465 and 1450 cm^–1^ with the vibrations of the CH groups in lipids (Bhat, 2013; Lecellier et al., 2014; Haneef et al., 2017; Kosa et al., 2018), it is worth noting that the interval between 1500 and 1300 cm^–1^ is often termed as the “mixed” region. Besides lipids, vibrations of functional groups bound in polysaccharides, proteins and triterpene compounds may be detected in the considered interval (Meenu and Xu, 2019; Saif et al., 2021). The majority of absorption at 1430 cm^–1^ was probably caused by C–O–H bending in polysaccharides (Song et al., 2015; Meenu and Xu, 2019), but some contributions from the COO^–^ group in carboxylic acids cannot be ruled out (Max and Chapados, 2004; Stuart, 2004).

In the case of the *Chlorella* sp. spectrum there is a relatively broad peak at 1397 cm^–1^, which can be associated with asymmetric stretching of COO^–^ (van Hoogmoed et al., 2003) and at the same time with the symmetric bending of CH in the CH_2_ and CH_3_ groups, respectively (D’Souza et al., 2008). A smaller peak at 1377 cm^–1^ can be assigned to symmetric bending of the aliphatic CH_3_ (Kosa et al., 2018; Meenu and Xu, 2019). Haneef et al. (2017) associated the absorption around 1375 cm^–1^ with the CH bending of chitin molecules in the fungal mycelium. In our case, the peak was present only in the *A. niger* spectrum, which supports the assumption that it was caused by chitin. The relatively sharp absorption at 1312 cm^–1^ was probably caused by the C–O stretching of carboxylic acids (Meenu and Xu, 2019) and/or the asymmetric C–N–C stretching of aromatic amines (Stuart, 2004; Silverstein et al., 2005).

The peaks at 1250 (*A. niger*) and 1242 cm^–1^ (*Chlorella* sp.), respectively, can in both cases be linked to the amide III band, where the absorption is associated with CN stretching, NH bending and CO in-plane bending (López-Lorente and Mizaikoff, 2016). It is probable that the absorption in this region was also partially caused by asymmetric P=O stretching in nucleic acids (Haneef et al., 2017) and phospholipid substances (Kosa et al., 2018). From a comparison of the spectra it follows that the peak around 1250 cm^–1^ was more intense in the case of *Chlorella* sp. than in *A. niger*. This suggests that the absorption was caused mainly by proteins and also partially by phospholipids, as their portion in the biomass is higher in comparison to nucleic acids (especially in *Chlorella* sp.).

The majority of the absorptions detected between 1200 and 900 cm^–1^ originate from the vibrations of carbohydrates. Specific functional groups and related frequencies include stretching vibrations of the COC (1150 cm^–1^), CO (1075 cm^–1^), and CC (1052 or 1034 cm^–1^) groups (Bhat, 2013; Baeva et al., 2019). In addition to carbohydrates, the C–O stretching of alcohols and sulfoxides occur at 1053 and 1034 cm^–1^, respectively (Rahman et al., 2014). However, the concentration of these substances in the biomass of both organisms is probably significantly lower in comparison to carbohydrates. Although the spectra of the *A. niger* and *Chlorella* sp. biomass were interpreted separately in this section, the adsorption experiments were carried out using the “mixture” of these two microorganisms. The spectrum of this this sample (without adsorbed Ni) is shown in Figure 5C.

### Functional Groups Involved in the Adsorption of Ni

**FIGURE 6:**
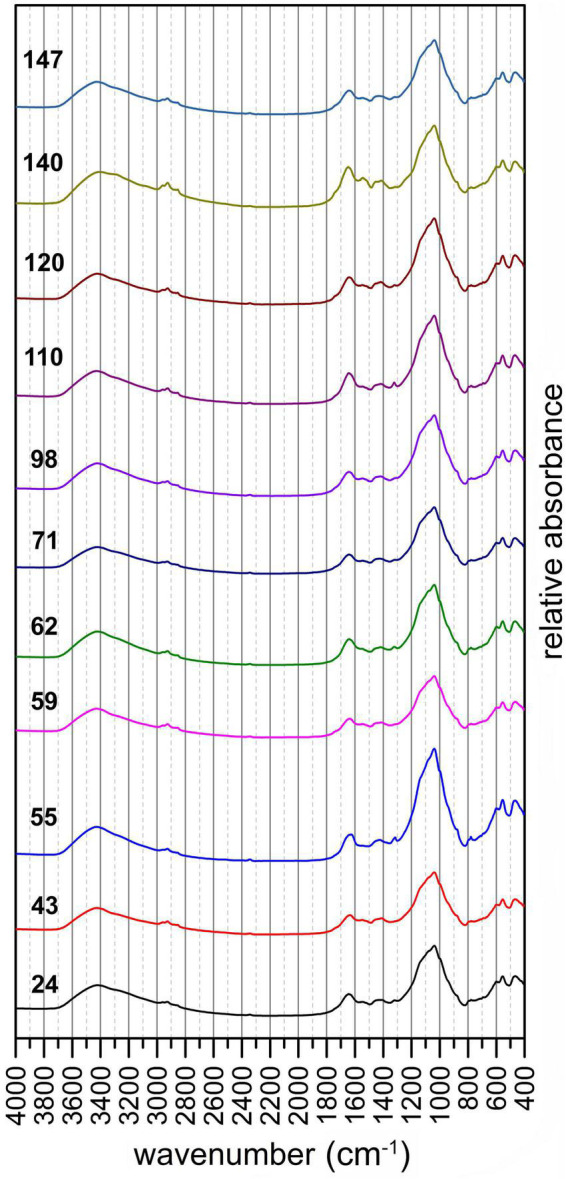
FTIR spectra of *Chlorella* sp./*Aspergillus niger* pellets after the adsorption experiments (values on the left indicate the amount of adsorbed Ni).

The interaction between the microbial biomass and the Ni substrate was assessed on the basis of a correlation spectrum. In the spectrum each value expresses the collinearity between the absorbance detected at certain wavelength and the amount of Ni retained by the sample. This approach enables better identification of the sorption sites in comparison to simple visual observation of the spectra. This follows from Figure 6, where the spectra are sorted according to the concentration of adsorbed Ni. It is hard to identify the change in absorbance or the shift in peak positions in the plotted spectra which would be related to concentration of Ni in the sample. Among the few visible changes in the spectra is the increase in absorbance between 1600 and 1400 cm^–1^, which was at the same time accompanied by a higher amount of adsorbed Ni. In this regard, the correlation spectrum is more informative, as it indicates which functional groups are involved in the sorption of Ni.

**FIGURE 7:**
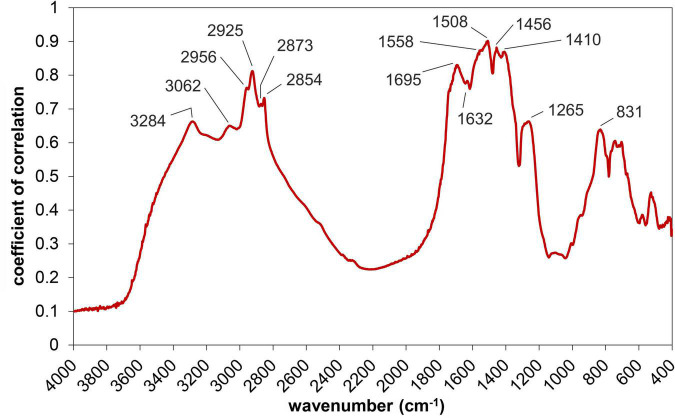
Correlation spectrum expressing collinearity between the amount of adsorbed Ni and the absorbance detected in the mid-infrared region (*r* > 0.6 corresponds to the *P* < 0.05 significance level).

The significance of the correlation (*P* < 0.05) was exceeded at frequencies characteristic for various organic groups (Figure 7). The first two peaks at the margin of the displayed spectral range, around 3284 and 3062 cm^–1^, may be assigned to stretching vibrations of the NH group in amides (Parker, 1971; van Hoogmoed et al., 2003; Forfang et al., 2017). However, the origin of the 3284 cm^–1^ peak may also be associated with OH stretch due to the formation aquo-metal complexes (Zhang et al., 2021). The four adjacent peaks, within the 2960 and 2850 cm^–1^ interval, represent the different stretching vibrations of aliphatic CH groups, specifically: methyl symmetric CH stretching at 2960 cm^–1^, methylene asymmetric CH stretching at 2930 cm^–1^, methyl asymmetric CH stretching at 2870 cm^–1^, and methylene symmetric C–H stretching at 2850 cm^–1^ (Stuart, 2004). A significant correlation between absorbance and the content of adsorbed Ni was also observed at 1695 and 1632 cm^–1^, respectively. The more apparent peak at 1695 cm^–1^ is due to the stretching vibrations of the C=O group, which is a part of various organic structures, including carboxylic acids (COOH), amides (CONH) or esters (COOR). On the other hand, the smaller peak with a maximum around 1630 cm^–1^ probably represents the C=O stretching vibration of a dissociated COO^–^ group (Antisari et al., 2011; Kang et al., 2011).

The correlation between absorbance and the content of adsorbed Ni showed higher values in the amide I and II bands, which correspond with the intervals of 1710–1600 and 1580–1520 cm^–1^, respectively (Lorenz-Fonfria, 2020). As was mentioned in the previous section, absorbance in amide I band is associated with the symmetric stretching of C=O and C–N together with N–H bending, whereas in case of the amide II band, the absorbance is indicative mainly of C–N stretching and N–H bending vibrations (Lecellier et al., 2014).

Although the correlation spectrum did not show peaks with maxima at 1650 or 1600 cm^–1^, the *r* values at these wave numbers clearly exceeded the significance level (*P* < 0.01). The mentioned frequencies are associated with vibrations of aromatic carbon (Rahman et al., 2014; Meenu and Xu, 2019). Some amino acids (and their metabolites), which are synthesized by algae and other microorganisms, contain an aromatic ring in their structure (Żyszka-Haberecht et al., 2019). Aromatic structural units are also bound in various pigments, some of which (melanins) are present in fungal mycelium (Rahman et al., 2014). It cannot be ruled out that the mentioned substances contributed to the sorption of Ni by the microbial biomass.

A significant correlation between adsorbed Ni and absorbance was observed at 1558 and 1508 cm^–1^, respectively. Both peaks are within the amide II interval, where IR absorption is caused by the combination of N–H bending and C–N stretching vibrations. In addition, the band around 1558 cm^–1^ was also associated with asymmetric stretching of the COO^–^ group of amino acids (Barth, 2000, 2007), carboxylic acids and their salts (Max and Chapados, 2004). This suggests that carboxyl groups can effectively contribute to Ni adsorption. The coefficient of correlation reached the highest value (0.9) at 1508 cm^–1^. In a study of a similar nature, where the removal of Cd from water by cyanobacterial biomass was assessed, Bon et al. (2021) assigned the IR adsorption at 1510 cm^–1^ to N–H bending. Besides the mentioned C–N stretching and N–H bending, vibrations of aromatic carbon may be detected at frequencies around 1508 cm^–1^, which means that various compounds containing an aromatic ring cause the absorption at the given wave number. In a number of works dealing with the composition of soil organic matter, the peak around 1510 cm^–1^ was associated with the stretching vibrations of aromatic carbon (Haberhauer and Gerzabek, 1999; Pietikäinen et al., 2000; Jiménez-González et al., 2019). Similar findings are reported in studies on the analysis of plant materials, where absorption at 1515 cm^–1^ indicates valence vibrations of aromatic C in phenolic compounds (Heredia-Guerrero et al., 2014). It is worth noting that Barth (2007) associated the band around 1510 cm^–1^ with the detection of amino acids containing an aromatic ring in their structure.

The correlation between absorbance and the content of adsorbed Ni also showed a maximum at 1456 cm^–1^. This peak probably represents the bending of CH_2_ groups in lipids, aliphatic structures (Mayers et al., 2013; Lecellier et al., 2014; Kosa et al., 2018), and polysaccharides (Meenu and Xu, 2019). The adjacent peak at 1410 cm^–1^ may be attributed to the asymmetric stretching of the COO^–^ group (van Hoogmoed et al., 2003). Even though the correlation culminating around 1265 cm^–1^ reached lower values in comparison to the IR bands mentioned above, the *P* < 0.05 significance threshold was exceeded. This band may be associated mainly with amide III vibrations, which consists of CN stretching, NH bending and CO in-plane bending (López-Lorente and Mizaikoff, 2016). Besides amides, IR absorption in this region is caused by asymmetric stretching of the P=O group in nucleic acids and phospholipids (Mayers et al., 2013; Lecellier et al., 2014; Kosa et al., 2018).

Absorption sites for Ni were found to provided mainly by amide, esters and lipidic structural units, whereas carbohydrates probably played a less important role in the sorption of Ni. This follows from the correlation between absorbance and the content of adsorbed Ni. In addition to the mentioned functional groups and structural moieties, it is also possible that other components of microbial biomass contributed to Ni adsorption, for example, amines or compounds containing an aromatic ring. On the other hand, the *r* values were mostly insignificant in the frequency interval characteristic for the vibrations of carbohydrates (between 1200 and 900 cm^–1^). It should be noted that carbohydrates also cause absorption in a relatively broad region above 3000 cm^–1^, where OH groups are detected. However, interpretation of the peaks between 3800 and 3000 cm^–1^ is often ambiguous, as the absorbance in this interval is strongly affected by the water content in the sample. Although we dried all samples before FTIR measurements, it is difficult to prevent the absorption of water from the atmosphere by sample material, which proceeds quite rapidly.

The following section compares our findings with the results of other works assessing biosorption. Based on the peak position shift, Kang et al. (2011) and Guarín-Romero et al. (2019) both suggested that mainly OH and HN groups are involved in the metal uptake by the algal biomass. This is in general accordance with the results presented here, as we observed that the amount of adsorbed Ni and absorbance in bands of OH and NH vibrations show significant correlation. Rahman et al. (2014), who studied the adsorption of metal ions on the surface of *Trichoderma* presented similar findings. They found that the adsorption of metal caused changes in the FTIR spectra mainly in the 3800–3000 and 1600–1700 cm^–1^ intervals, where the vibrations of hydroxyl, carbonyl, carboxyl, and amide groups are detected, respectively. Besides the mentioned bands and functional groups, we also observed that other structural units may be involved in biosorption, which follows from the significant correlation of the 1600–1400 cm^–1^ interval. A substantial part of this region corresponds with the amide II band, but at lower wave numbers the vibrations of the other groups manifest, including CH and COO^–^ groups or an aromatic ring. Our observations are in relative accordance with the findings of Pradhan et al. (2007), who reported an increase of absorbance at 1550, 1538, and 1513 cm^–1^, respectively, due to a change of the C=O stretching after Ni sorption on *Microcystis* capsules. We observed a significant correlation between absorbance detected at mentioned frequencies and the content of adsorbed Ni. The calculated *r* values were in this case above 0.87 (*P* < 0.001), and the correlation reached a maximum at 1508 cm^–1^.

Although the value of the correlation coefficient and the peak positions indicate which functional groups are involved in the sorption of Ni, some aspects of this approach should be considered. A significant positive correlation between the content of adsorbed Ni and the absorbance detected at specific frequency does not prove that the group itself is directly involved in the sorption of Ni. Even if we exclude the overlapping of the respective spectral bands (which is a factor that is hard to eliminate), it is still possible that the considered functional group is part of a molecule that may be involved in the sorption process, but the group itself is not. In this study we observed a significant positive correlation in the case of IR bands associated with several functional groups, including OH, CH, COO^–^, C=O, and NH groups, respectively. However, the presented FTIR results did not prove that all the mentioned groups were actively participating in the adsorption of Ni. It is possible that the structural units (or molecules) of the microbial biomass which were involved the adsorption of Ni contain the mentioned functional groups but that some of them (CH groups) did not participate in the adsorption. On the other hand, there are different adsorption mechanisms. Besides chemical bonding, which takes place between Ni cation and negatively charged functional groups (such as COO^–^), weaker electrostatic attraction plays a role in biosorption, as well (Ren et al., 2021). In such a case, less reactive structural units, including non-polar CH groups, also take part in the retention of Ni. These factors probably contributed to the significant positive correlation that was observed between the content of adsorbed Ni and absorbance in the bands of CH vibrations (and possibly also vibrations of an aromatic ring).

## Conclusion

This study showed that microbial consortium composed of algal biomass and fungi is capable of effectively reducing the Ni content in ash waste material. During the experiment, organic acids produced by *A. niger* disrupted the cell wall of the microalgae *Chlorella* sp.; the chloroplasts disintegrated and the chlorophyll concentration decreased, resulting in a change of color. Organic matter, which is a source of nutrients for *A. niger*, was released into the environment. In addition to the accumulation by the microscopic fungi (*A. niger*), biosorption mechanisms, such as ion exchange, microprecipitation and interaction of metal ions with functional groups of the algal culture surface of *Chlorella* sp., also took place concurrently. FTIR spectroscopy helped to identify the functional groups involved in the adsorption of Ni from the solid waste substrate by the *Chlorella* sp./*A. niger* biomass. These groups include hydroxyl, aliphatic, carbonyl, carboxyl, and amide structural units. The observed correlations between absorbance and adsorbed Ni content indicate that, in addition to polar (C=O) and negatively charged (COO^–^) groups, aliphatic or aromatic structures may also be involved in sorption due to the weaker electrostatic attraction. The correlation between absorbance and Ni content reached a maximum in amide II band, where vibrations of the C=O, C–N, and N–H groups are detected. This suggests that during Ni adsorption, a significant portion of the adsorption sites is provided by the structural units of proteins in the biomass of *Chlorella* sp. and/or *A. niger*. It should be added that, despite the presented findings, it is relatively difficult to clearly identify the most important groups involved in adsorption based on the results of FTIR spectroscopy. This is partly due to the variable and complex structure of proteins. Another factor is the overlapping of the spectral bands in which the respective functional groups are detected. This also applies to the band of the highest correlation (1560–1500 cm^–1^), where, besides the mentioned amide groups, vibrations of aromatic carbon may also be detected. Based on the observed correlations and results reported in previous works, it can be concluded that various organic structures are involved in the adsorption of heavy metal cations, and a strict attempt to identify “the most important” may lead to oversimplification of the true nature of the adsorption processes. Experiments carried out in this study demonstrate cost-effective application of microbial consortium in decontamination of waste ash material. The results suggest that proper combination of fungi and algae, as well as their simultaneos application may result in synergic mechanism of Ni adsorption. Lowering the concentration of risk element in the ash is important not only in the context of safe disposal, but also for purpose of recovery and separation of metals. This pertains especially to Ni, which is widely used in the industry and up to date its recyclation has been limited.

## Data Availability Statement

The original contributions presented in the study are included in the article/Supplementary Material, further inquiries can be directed to the corresponding author.

## Author Contributions

AŠ: isolation of *Aspergillus niger* strain and preparation of fungal pellets. AT: work with *Chlorella* sp.. AŠ and AT: design and organization of the experiments. IŠ: FTIR analyses. SN: preparation of photo documentation. All authors prepared the manuscript, read, and agreed to the published version of the manuscript.

## Conflict of Interest

The authors declare that the research was conducted in the absence of any commercial or financial relationships that could be construed as a potential conflict of interest.

## Publisher’s Note

All claims expressed in this article are solely those of the authors and do not necessarily represent those of their affiliated organizations, or those of the publisher, the editors and the reviewers. Any product that may be evaluated in this article, or claim that may be made by its manufacturer, is not guaranteed or endorsed by the publisher.
