# Supplementary figures and images for: Experimental Treatment of Hazardous Ash Waste by Microbial Consortium Aspergillus niger and Chlorella sp.: Decrease of the Ni Content and Identification of Adsorption Sites by Fourier-Transform Infrared Spectroscopy
Source: Front Microbiol. 2021 Dec 7;12:792987. doi: 10.3389/fmicb.2021.792987 (PMC8689076; doi:10.3389/fmicb.2021.792987)

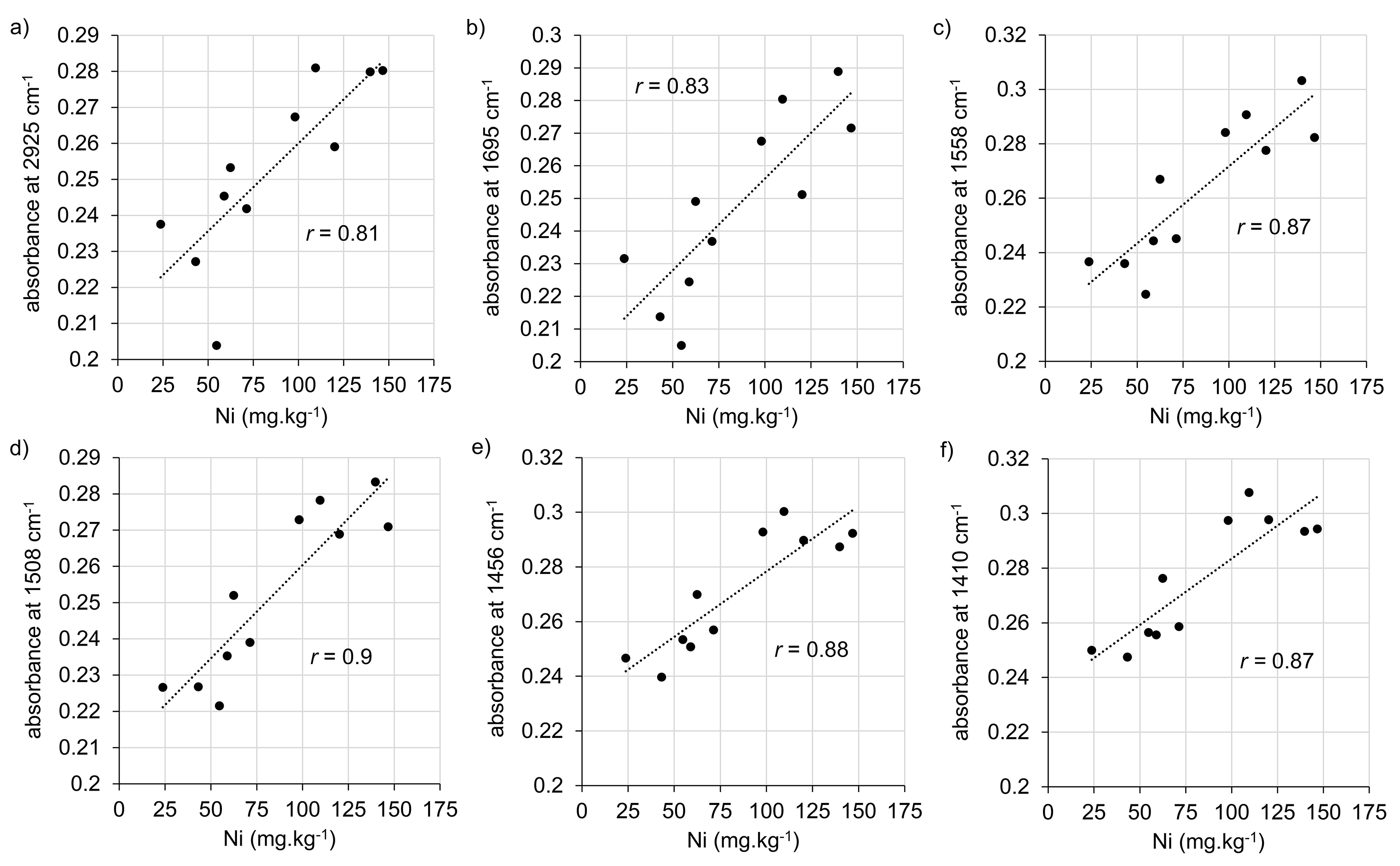

Supplement: Supplementary file 1 [file Image_1.JPEG]
